# The Role of Different Medical Therapies in the Management of Adenomyosis: A Systematic Review and Meta-Analysis

**DOI:** 10.3390/jcm13113302

**Published:** 2024-06-04

**Authors:** Giulia Galati, Gianfilippo Ruggiero, Alice Grobberio, Oriana Capri, Daniela Pietrangeli, Nadia Recine, Michele Vignali, Ludovico Muzii

**Affiliations:** 1 Department of Maternal and Child Health and Urology, Sapienza University, 00161 Rome, Italy; giulia.galati@uniroma1.it (G.G.); gianfilippo.ruggiero@uniroma1.it (G.R.); alice.grobberio@uniroma1.it (A.G.); oriana.capri@uniroma1.it (O.C.); daniela.pietrangeli@uniroma1.it (D.P.); nadia.recine@uniroma1.it (N.R.); 2 Department of Biomedical Sciences for Health, University of Milan, 20133 Milan, Italy; michele.vignali@unimi.it

**Keywords:** adenomyosis, medical therapy, dysmenorrhea, menorrhagia

## Abstract

**Background/Objectives**: Adenomyosis is a benign condition characterized by the presence of endometrial tissue within the myometrium. Despite surgery being a valuable approach, medical options are considered as the first-line approach and have been investigated in the treatment of adenomyosis, although strong evidence in favor of these is still lacking. This study aims to gather all available data and determine the effectiveness of the aforementioned medical options in patients with associated pain and not currently seeking pregnancy, both in comparison to placebo and to one another. **Methods:** For this study, PubMed and EMBASE were used as data sources, searched up to January 2024. A systematic review and meta-analysis were performed in accordance to guidelines from the Cochrane Collaboration. The primary outcomes investigated were changes in dysmenorrhea, quantified by means of VAS scores, HMB in terms of number of bleeding days, and changes in uterine volume determined at ultrasound. Twelve eligible studies were selected. **Results:** The results highlighted that dienogest yields a reduction in dysmenorrhea that is significantly superior to that of the rest of the medical treatments investigated (*p*-value of <0.0002). On the other hand, GnRH agonists seem to play a more prominent role in reducing uterine volume (*p*-value of 0.003). While it was not possible to determine which medical treatment better decreased the number of bleeding days, it was observed that COC performed significantly worse than the other treatments studied (*p*-value of 0.02). **Conclusions:** While this meta-analysis provides valuable insights in the comparative efficacy of different treatments, the paucity of relevant studies on the topic might impact the reliability of some of the conclusions drawn.

## 1. Introduction

Adenomyosis is an estrogen-dependent, benign uterine disease that is diagnosed when the myometrium is invaded by endometrial glands and stroma [1]. Adenomyosis has traditionally been reported to affect women between the age of 40 and 50 years as well as multiparous patients. Although approximately one-third of patients are asymptomatic, typically adenomyosis symptoms include heavy menstrual bleeding (HMB), dysmenorrhea, chronic pelvic pain, and infertility, severely impacting the quality of life of these patients [2].

HMB occurs in approximately 50 to 60 percent of patients with adenomyosis and may be related to the increased surface of the enlarged uterus, an overexpression of inflammatory mediators in the adenomyotic tissue, or contractile dysfunction of the uterine smooth muscle as a result of the ectopic location of the endometrium [3].

Dysmenorrhea occurs in approximately 25 to 80 percent of patients and may be due to increased bleeding and swelling of endometrial islands in the myometrium [2].

The pathogenesis and etiology of adenomyosis are still uncertain. Traditionally, epidemiological data suggest that a high number of deliveries, spontaneous and induced abortions, chronic endometritis, and hyperestrogenism could be possible risk factors for adenomyosis [4]. Furthermore, with an estimated prevalence of 46%, adenomyosis is becoming increasingly more frequent in adolescents, too [5].

Traditionally, the diagnosis of adenomyosis was made retrospectively and based solely on histologic assessment of hysterectomy specimens. However, the diagnosis is now made by imaging-based criteria using transvaginal ultrasonography (TVUS) and/or magnetic resonance imaging (MRI), which has resulted in a greater understanding of the disease, its prevalence, effect on younger patients and patients with reproductive dysfunction, and treatment options. In fact, studies utilizing imaging diagnosis suggest that adenomyosis can frequently be detected at ages younger than 40 years (prevalence rates of 20 to 35 percent) [6].

To date, there are several medical therapeutic strategies to treat this condition, but few studies confirm their effectiveness with robust evidence. Similarly to endometriosis [7], the most widely used drugs are the following: levonorgestrel intrauterine systems (LNG-IUS), which, given their direct action on the uterus, low systemic levels of steroid hormones, and long-acting user-independent administration, improve both adenomyosis-associated HMB and dysmenorrhea [8,9]; combined oral contraceptives (COC) that, in decreasing menstruation and producing a pseudogestational state, cause endometrial atrophy [10]; and dienogest, a selective synthetic oral progestin that uniquely combines the pharmacological properties of progesterone and a 19-norprogestin derivative [11].

Furthermore, gonadotropin-releasing hormone analogues (GnRHa) can also be administered, which produce a constant hypoestrogenic status [12,13], and selective progesterone receptor modulators (SPRMs), such as ulipristal acetate (UPA).

Certainly, the treatment of adenomyosis also involves surgical procedures such as hysterectomy, adenomyomectomy, embolization of the uterine arteries, laparoscopic radiofrequency ablation, and transcervical radiofrequency ablation. However, the aim of this study is limited to the evaluation of the efficacy of medical therapeutic strategies as compared to placebo or to other medical treatments to improve the clinical symptoms or ultrasound features of adenomyosis. The evaluation of medical treatment of adenomyosis before in vitro fertilization in infertile patients was not among the scope of the present review.

## 2. Materials and Methods

The present systematic review and meta-analysis was completed according to the methodologic standards of the Cochrane Handbook [14] and the Preferred Reporting Items for Systematic Reviews and Meta-Analyses guidelines (PRISMA).

The study protocol was registered online through an International Prospective Register of Systematic Reviews (PROSPERO number: CRD42023442081). Since published data were used, this study was exempt from Institutional Review Board approval.

### 2.1. Selection Criteria

The present systematic review and meta-analysis included randomized clinical trials (RCTs), prospective studies, or retrospective controlled studies investigating the effect of all proposed medical therapies for adenomyosis on dysmenorrhea, HMB, and uterine volume. In particular, all research studies had to include women with a diagnosis of adenomyosis based on ultrasound features (one or more of the following: globular uterine enlargement, asymmetrical myometrial thickening, myometrial cysts, echogenic striations, myometrial lesions, or irregular endometrial/myometrial border) [15]. Changes in dysmenorrhea, HMB, and uterine volume were recorded after a preplanned study period.

Inclusion criteria of studies were (1) articles in the English language; (2) articles written within the last 15 years; (3) randomized clinical trials (RCTs), prospective studies, or retrospective controlled studies; (4) articles comparing at least two types of medical treatment or a medical treatment to placebo or no treatment; and (5) evaluation of at least one outcome of interest.

### 2.2. Search Strategy and Study Selection

An extensive literature search was conducted by the different authors to identify all studies on the topics of adenomyosis and its medical treatments published until January 2024. The electronic databases used in the article selection process were the following: EMBASE, PubMed, and Cochrane Collaboration. The following Medical Subject Heading terms were used for retrieval: “adenomyosis treatment”, “dienogest”, “combined oral contraceptives”, “gonadotropin-releasing hormone analogues”, “levonorgestrel intrauterine system”, “selective progesterone receptor modulators”, AND “adenomyosis medical therapy”. The search strategy is described in detail in Supplementary Data File S1, available online. In the attempt to identify further published, unpublished, and ongoing trials, we searched trials and research registries. Moreover, the reference lists of reviews and relevant articles were screened by hand to identify additional eligible publications. Letters, editorials, and case reports were excluded from this review.

All steps of study selection were performed independently by two reviewers. A broadly inclusive search was conducted initially, followed by a subsequent restriction for studies on women with symptomatic adenomyosis undergoing medical treatment during the title/abstract review process. Then, the full texts of preliminary selected articles were reviewed by the two authors separately to screen out duplicate and irrelevant articles. Subsequently, studies were excluded in case of incomplete data or incompatible interventions.

### 2.3. Outcomes and Data Extraction

Outcomes that are particularly concerning for patients in this context were selected.

#### 2.3.1. Primary Outcomes

(1) Dysmenorrhea: Evaluation of dysmenorrhea using standardized measures (10-point visual analogue scale (VAS), with conversion to a 10-point scale in case studies reporting a 1–100 mm scale) to score the symptom intensity from baseline to follow-up period;
(2) HMB: Assessed at baseline and at follow-up after the treatment by compiling a menstrual diary, taking into account the number of bleeding days, or by assessing the volume of blood lost per menstruation;
(3) Changes in uterine volume: Determining ultrasonographically the volume of the uterus at baseline and at a time interval after the beginning of therapy.

The articles included had to present numerical results on the effects of medical treatments, avoiding the comparison with surgical interventions.

#### 2.3.2. Data Extraction

The data used in the statistical analysis were taken directly from the results tables depicted in the included articles. For the studies that failed to show their results expressed as mean and standard deviation in the text or in the tables but rather presented them only as graphs, attempts were made to contact the corresponding authors and obtain numerical data. If treatment dosage differed within the same study, the patient group receiving the same dosage as that of the same medication in other studies was selected for our statistical analysis. If studies compared more than two treatments, the arm with the least number of patients was excluded from the statistical analysis.

### 2.4. Risk of Bias

The risk of bias was appraised independently by two reviewers, according to the guidelines published in the Cochrane Handbook for Systematic Reviews of Interventions regarding the following aspects: the generation of random sequences, concealment of allocation, blinding of participants and implementers, blinding of implementation to outcome evaluation, completeness of outcome data, selective outcome reporting, and other sources of bias. The risk of bias in observational studies was assessed by the ROBINS-I tool (Supplementary Materials—Table S1).

### 2.5. Statistical Analysis

Statistical analysis was carried out extracting relevant data from the articles selected. Data were pooled using RevMan software (Review Manager version 5.4; the Cochrane Collaboration, Copenhagen, Denmark). Dichotomous outcomes from each study were expressed as odds ratios (OR) with a 95% confidence interval (CI). Continuous outcomes were expressed as standardized mean differences (SMD). Heterogeneity between studies was reported with the I^2^ statistic. A DerSimonian–Laird random-effect meta-analysis model was used at meta-analysis if any heterogeneity was detected, whereas a fixed-effects model was used if no heterogeneity was identified. A value of *p* < 0.05 was considered statistically significant.

## 3. Results

### 3.1. Study Selection

An initial search performed by looking up the keywords “adenomyosis” and “medical treatment” yielded a total of 915 studies. After a primary screening, 780 of them were removed for not meeting the inclusion criteria previously outlined and 92 of them for being written in languages other than English. Of the 43 articles screened, further selection was performed based on the type of study and statistical analysis performed. The final number of studies that were eligible and thus included in this meta-analysis was 12. Information about the characteristics of these studies is included in Table 1.

**Figure 1:**
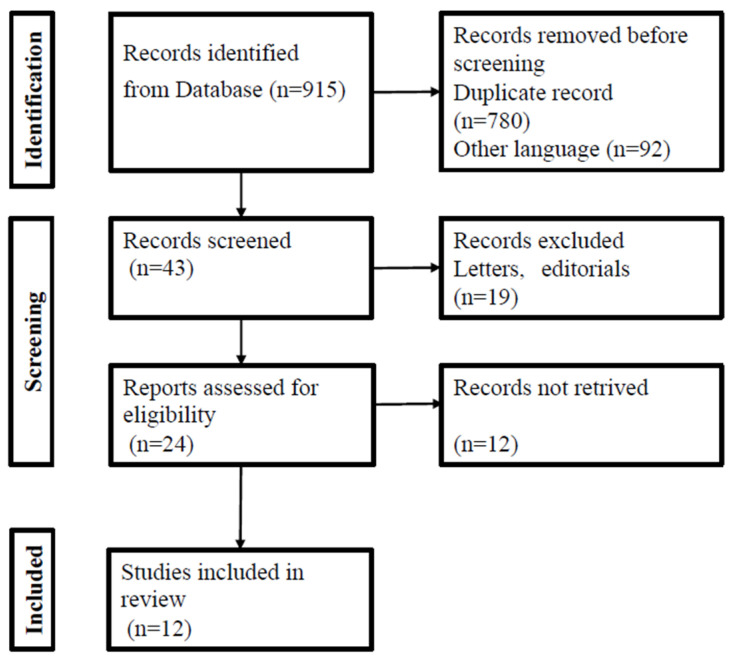
PRISMA flowchart for study identification and inclusion/exclusion.

The flow diagram for the search and selection of the articles is depicted in Figure 1.

### 3.2. Study Characteristics and Quality of Evidence

Out of the studies selected, only eight were RCTs [13,16,19,21,22,24,25,26], while the remaining consisted of three prospective studies [17,18,23] and one retrospective study [20]. Dienogest was investigated in seven of the studies analyzed (of which four were RCTs), LNG-IUS in four (three RCTs), GnRH agonists in four (one RCT), aromatase inhibitors in one RCT, combined oral contraceptives (COC) once daily for 21 days followed by a 7-day pill-free interval in three (two RCTs), UPA in one RCT, and mifepristone in one RCT.

The RCT by Shaaban et al. [16] included 62 women suffering from diffuse adenomyosis complaining of menorrhagia and dysmenorrhea. LNG-IUS or gestodene-containing COC were administered for six months, and VAS, blood loss, and uterine volume were measured during a follow-up period of 6 months. Badawy et al. [13], on the other hand, compared aromatase inhibitors and GnRH agonists in their RCT, recruiting a total of 32 women and assessing the decrease in uterine volumes at different time intervals (4 weeks, 8 weeks, and 12 weeks) during the therapy and then calculating a mean value. In the RCT by Osuga et al. [19], 67 women suffering from adenomyosis were prescribed either dienogest 2 mg/day orally or placebo for 16 weeks, using VAS to assess the change in intensity of painful symptoms. Hassanin et al. [21], in their RCT, observed the changes in VAS scores after 6 months of treatment with either dienogest or gestodene-containing COC while also reporting changes in uterine volume and bleeding patterns. In the RCT by Capmas et al. [22], a total of 40 women were given either UPA or placebo in a 3:1 ratio, and the pictorial blood loss assessment score was used to determine the decrease in blood loss. Guo et al. [24], in their RCT, compared LNG-IUS and DNG in 117 women. VAS scores, uterine volume, endometrial thickness, serum CA 125 level, estradiol, follicle-stimulating hormone, and luteinizing hormone were considered during 36 months. In the RCT by Che et al. [25], a total of 126 women were treated for 12 weeks with mifepristone at a dose of 10 mg per day or placebo, and valued remission of dysmenorrhea, reduction in uterine volume, change in menstrual blood loss, increased level of hemoglobin in patients with anemia, serum CA 125 level, and platelet count were measured. Lastly, the RCT of Choudhury et al. [26] compared the treatment with LNG-IUS versus DNG in 84 women with symptomatic adenomyosis, analyzing reduction in painful symptoms after 12 weeks of treatment measured by VAS score, changes in menstrual blood loss (MBL), and improvement in quality of life (QoL).

VAS scores were used to calculate changes in dysmenorrhea in eight of the selected studies, while three of the studies quantified this outcome by determining how many people reported painful menstrual cycles after therapy. Uterine volume was derived ultrasonographically in 10 of the studies. In two studies, uterine volumes were not assessed. Data regarding changes in bleeding patterns were only reported in six studies, with four of them quantifying it as the number of bleeding days and one of them calculating the blood loss in mL and another as a pictorial blood loss assessment score value. Since there were some inconsistencies in the modality in which each of the studies quantified their primary outcomes, some of the studies selected were not included in the design of the forest plots. Based on the ROBINS-I tool, we identified 12 studies with moderate risk of bias.

### 3.3. Study Outcomes

#### 3.3.1. Changes in Dysmenorrhea

Six forest plots were created, each comparing a particular regime of medical treatment to all the other options investigated in the selected studies. Some of the studies reported data acquired at different time intervals, thus allowing us to investigate the effects of the treatments in both the short and long term. We arbitrarily considered as short term any follow-up period of up to six months, while a follow-up period between 12 and 24 months would be considered long term. With the available short-term data, we were able to individually compare treatment with LNG-IUS, DNG, COC, GnRHa, and placebo to all the other treatment options.

**Figure 2:**
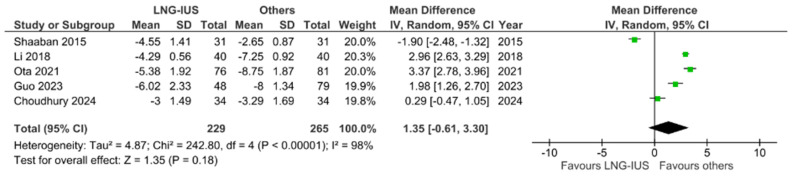
Forest plot—changes in dysmenorrhea: LNG-IUS vs. others in the short term [16,18,23,24,26].

**Figure 3:**
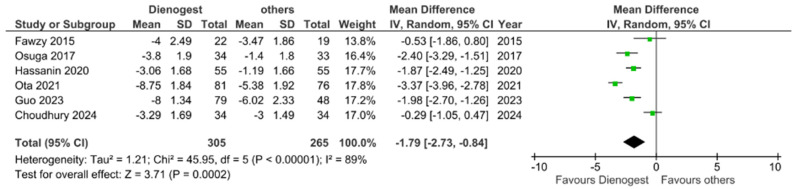
Forest plot—changes in dysmenorrhea: DNG vs. others in the short term [17,19,21,23,24,26].

As shown in Figure 2, LNG-IUS did not reduce VAS in a manner significantly different from that caused by the other treatments investigated [16,18,23,24,26]. On the other hand, according to the included studies, the difference in outcomes between DNG and all other treatments is indeed significant. With a *p*-value of 0.0002, our study favors the use of DNG to reduce dysmenorrhea over that of any of the other treatment choices (Figure 3) [17,19,21,23,24,26].

**Figure 4:**
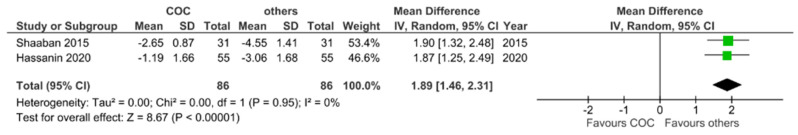
Forest plot—changes in dysmenorrhea: COC vs. others in the short term [16,21].

Figure 4 shows how COC fare with respect to other treatments in the reduction in dysmenorrhea. Our statistical analysis showed that there is a significant difference in favor of treatments other than COC, which in this particular case were LNG-IUS and DNG [16,21].

We also analyzed the effects of GnRHa and placebo on dysmenorrhea, failing to highlight a significant advantage of these two managements over any other treatment (Supplemental Materials Figure S1 [17,18] and Figure S2 [19,22,25]). Regarding the comparison of placebo to other treatments, we acknowledge that the control group of the included studies was represented by therapies that are rarely used (UPA and mifepristone).

**Figure 5:**
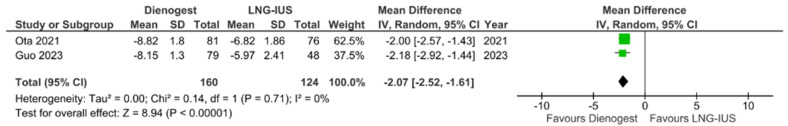
Forest plot—changes in dysmenorrhea: DNG vs. LNG-IUS in the long term [23,24].

In the long term, the studies available allowed a direct comparison between LNG-IUS and DNG. DNG showed superiority over LNG-IUS in dysmenorrhea reduction in the long term (Figure 5) [23,24].

#### 3.3.2. Changes in Uterine Volume

The changes in uterine volume at ultrasound examination were reported and analyzed in nine studies with follow-up periods of up to 6 months. Supplemental Materials Figure S3 is a forest plot representing the non-significant changes in volume yielded by LNG-IUS versus any other treatment [16,18,24]. Similarly, the forest plots in Figure S4 [17,19,20,21,24] and Figure S5 [16,20,21] that compare DNG and COC, respectively, to all the other treatments fail to show any significantly different reduction in uterine volume.

**Figure 6:**
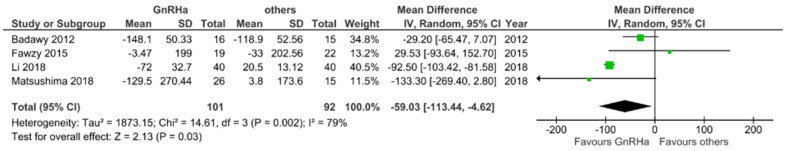
Forest plot—changes in uterine volume: GnRHa vs. others [13,17,18,20].

The forest plot in Figure 6 shows the differences in volume reduction between GnRHa and all the other treatments [13,17,18,20]. With a *p*-value of 0.03, this forest plot can thus conclude that GnRH agonists cause a statistically significant reduction in uterine volume.

#### 3.3.3. Changes in Bleeding Patterns

**Figure 7:**
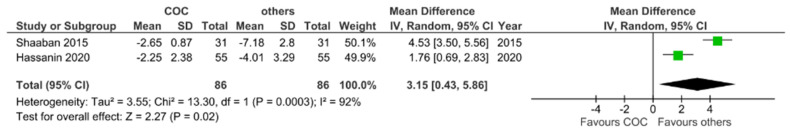
Forest plot—changes in bleeding patterns: COC vs. others [16,21].

The only three studies that reported the difference in number of bleeding days before and after therapy were those by Hassanin et al. [21], Ota et al. [23], and Shaaban et al. [16]. LNG-IUS and DNG did not perform significantly better or worse than other therapies in the reduction in the number of bleeding days (Supplemental Materials Figure S6 [16,23] and Figure S7 [21,23]). Our analysis favors the use of other treatments instead of COC as a means of reducing the number of bleeding days, as shown in Figure 7 [16,21]. The other two treatments included by Hassanin et al. and Shaaban et al. were DNG and LNG-IUS, respectively. With a significant *p*-value of 0.02, we can conclude that COC are less effective in reducing the number of bleeding days than the other treatments investigated. 

## 4. Discussion

The present systematic review and meta-analysis aimed at assessing the efficacy of different medical therapies in managing adenomyosis, focusing on symptoms and ultrasound characteristics.

Dysmenorrhea exhibited a statistically significant short-term reduction with the use of DNG compared to other medical treatments (OR −1.79, 95% CI −2.73 to −0.84, *p* = 0.0002). In the long term, our investigation was able to conclude that DNG performed significantly better than LNG-IUS (OR −2.07, 95% CI −2.52 to −1.61, *p* < 0.00001). However, no significant changes were observed following short-term LNG-IUS, GnRHa and placebo treatment versus any other treatment. It is of importance to note that the lack of a statistically significant results in the comparison of placebo to other treatments may depend on the therapies used in the control groups (UPA and mifepristone). On the other hand, our research showed that COC are less effective than LNG-IUS and DNG in reducing dysmenorrhea (OR 1.89, 95% CI 1.46 to 2.31, *p* < 0.00001).

Uterine volume was significantly reduced in the short term by GnRHa compared to other medical treatments (OR −59.03, 95% CI −113.44 to −4.62, *p* = 0.03), while it remained unchanged after LNG-IUS, DNG, or COC treatment.

In terms of uterine bleeding, a 6-month period of COC treatment demonstrated a significantly lower effectiveness in reducing menorrhagia compared to the other treatments investigated, namely DNG and LNG-IUS (OR 3.15, 95% CI 0.43 to 5.86, *p* = 0.02). DNG and LNG-IUS showed no superiority over one another in reducing the number of bleeding days.

The present study aligns with the existing literature on adenomyosis, emphasizing the challenges posed by symptoms such as dysmenorrhea, HMB, and uterine volume enlargement. Our findings contribute to the growing body of evidence regarding the effectiveness of specific medical therapies in addressing these symptoms. Notably, this study provides valuable insights into the comparative efficacy of different treatments, offering a nuanced understanding that can guide clinical decisions.

The current systematic review and meta-analysis aligns with and builds upon the existing literature on adenomyosis, contributing to the ongoing discourse surrounding the management of this estrogen-dependent uterine disorder. While approximately one-third of affected individuals remain asymptomatic, the symptomatic presentation is characterized by HMB, chronic pelvic pain, dysmenorrhea, AUB, infertility, and increased uterine volume, collectively impacting the quality of life [2].

Traditionally, the diagnosis of adenomyosis relied on retrospective histologic assessments of hysterectomy specimens [1]. However, advancements in imaging techniques such as TVUS and MRI have facilitated more accurate and non-invasive diagnosis, revealing a greater prevalence of adenomyosis, even in younger age groups [6].

The literature recognizes the multifaceted nature of adenomyosis, with HMB affecting approximately 50 to 60 percent of patients and painful menses reported in 25 to 80 percent of cases [2,3]. The underlying pathogenesis and etiology of adenomyosis remain uncertain, with epidemiological data suggesting potential risk factors such as a high number of deliveries, spontaneous and induced abortions, chronic endometritis, and hyperestrogenism [4].

In the realm of medical therapies, various strategies have been explored to address adenomyosis symptoms. Notably, LNG-IUS has garnered attention for its direct action on the uterus, low systemic levels of steroid hormones, and long-acting user-independent administration, effectively improving adenomyosis-associated HMB and dysmenorrhea [8,9]. COC, dienogest, GnRHa, and UPA have also emerged as widely used options, each exerting specific pharmacological effects [10,11]. This extensive review of the literature underscores the complex landscape of adenomyosis management, with various therapeutic options demonstrating variable efficacy. The present meta-analysis contributes to this body of knowledge by systematically evaluating the comparative effectiveness of different medical therapies, offering valuable insights into their respective impacts on dysmenorrhea, HMB, and uterine volume.

Several limitations should be acknowledged in interpreting the results. The meta-analysis primarily included only eight randomized clinical trials (RCTs), which might increase selection bias. Additionally, the lack of standardization in symptom assessment parameters and diagnostic criteria among the included studies could contribute to variability in results. The limited data for some treatments might have impacted the reliability of certain outcomes. Further research with a broader range of study designs and standardized outcome measures is warranted to enhance the robustness of the findings.

Despite the limitations, this systematic review and meta-analysis contributes to the field by providing a comprehensive overview of the relative efficacy of various medical therapies in adenomyosis. The evidence is generally consistent, and the study employed rigorous methodological standards, following the Cochrane Handbook and PRISMA guidelines [14,27]. The focus on dysmenorrhea, HMB, and uterine volume as primary outcomes enhances the relevance of the findings for clinicians managing adenomyosis patients.

## 5. Conclusions

In conclusion, this study underscores the heterogeneous responses of patients to different medical therapies for adenomyosis in patients not currently seeking pregnancy. The evaluation of medical therapies in infertile patients was not among the scope of the present review. DNG appears to be particularly effective in reducing dysmenorrhea, while COC seem to perform poorly in this regard. Furthermore, GnRHa show promise in reducing uterine volume compared to other treatments, and COC seem to be less effective in the management of menorrhagia. The conclusions drawn thus emphasize the need for more tailored therapeutic approaches. The above findings provide a foundation for personalized treatment protocols based on specific patient characteristics and symptomatology. Further research, including well-designed RCTs with standardized outcome measures, is essential to validate and expand upon these findings, ultimately advancing the management of adenomyosis and improving patient outcomes.

## Figures and Tables

**Table 1:** General characteristics of the included studied.

Author and Year	Country	Study Period	Study Design	Patients Number	Age Mean (Years)	Comparison	Follow Up	Outcomes
Badawy et al., 2012 [13]	Egypt	December 2005 to January 2010	Randomized clinical trial	32	AI 37 ± 3.44	Aromatase inhibitors/ GnRH agonists	4, 8, and 12 weeks	Dysmenorrhea, uterine volume
					GnRH agonists 35 ± 2.8			
Shaaban et al., 2015 [16]	Egypt	August 2013 to November 2014.	Randomized clinical trial	62	LNG-IUS 39.39 ± 4.43	LNG-IUS/COC	6 months	Dysmenorrhea, uterine volume, menstrual bleeding, increase in blood flow resistance
					COC 39.16 ± 3.21			
Fawzy et al., 2015 [17]	Egypt	May 2013 to November 2014	Prospective clinical trial	41	DNG 39.8 ± 4.3	Dienogest/GnRH agonists	16 weeks	Dysmenorrhea, uterine volume, menorrhagia, Hb (gm/dL), Ferritin (ng/mL)
					GnRH agonists 40.2 ± 5.7			
Li et al., 2017 [18]	China	February 2015 to February 2016	Prospective parallel-controlled study	200	GnRH agonists 36.28	GnRH agonists/LNG-IUS	3, 6, and 12 months	Dysmenorrhea, uterine volume
					LNG-IUS 40.45			
Osuga et al., 2017 [19]	Japan	August 2014 to June 2015	Randomized clinical trial	68	DNG 37.3 ± 7.9	Dienogest/placebo	16 weeks	Dysmenorrhea, uterine volume
					PL 37.4 ± 6.6			
Matsushima et al., 2018 [20]	Japan	August 2007 to July 2015	Retrospective cohort study	28	GnRH agonists 40.0 ± 6.1	GnRH agonists/COC/dienogest	16 weeks	Dysmenorrhea, uterine volume, Menorrhagia, CA125 (U/mL)
					COC 37.7 ± 5.3			
					DNG 38.9 ± 7.8			
Hassanin et al., 2020 [21]	Egypt	March 2019 to August 2020	Randomized clinical trial	97	COC 40.36 ± 3.73	COC/dienogest	6 months	Dysmenorrhea, uterine volume, ovarian volume, artery RI and PI
					DNG 39.96 ± 3.87			
Capmas et al., 2020 [22]	France	June 2016 to February 2018	Randomized controlled study, double-blind	40	UA 43 (37–45)	Ulipristal acetate/placebo	5, 9, and 13 weeks and 6 months	Dysmenorrhea, amenorrhea, anemia, quality of life
					PL 42.5 (39–47)			
Ota et al., 2021 [23]	Japan	January 2013 to December 2020	Prospective clinical trial	157	LNG-IUS 42.3 ± 4.2	LNG-IUS/dienogest	72 months	Dysmenorrhea, uterine volume, bone mineral density (BMD)
					DNG 41.4 ± 3.5			
Guo et al., 2023 [24]	China	May 2019 to June 2022	Randomized clinical trial	117	LNG-IUS 39.3 (5.2)	LNG-IUS/dienogest	36 months	Dysmenorrhea, uterine volume, CA125, endometrial thickness, FSH, LH
					DNG 39.7 (6.3)			
Che et al., 2023 [25]	China	May 2018 to April 2019	Randomized clinical trial	134	MF 40.2 [4.6]	Mifepristone/placebo	12 weeks	Dysmenorrhea, uterine volume, menorrhagia, anemia, CA125, platelet count
					PL 41.7 [5.0]			
Choudhury et al., 2024 [26]	India	June 2020 to August 2021	Randomized clinical trial	74	LNG-IUS 40.06 ± 6.95	LNG-IUS/dienogest	12 weeks	Dysmenorrhea, menstrual blood loss, quality of life
					DNG 40.97 ± 6.78			

AI, aromatase inhibitors; DNG, dienogest; PL, placebo; MF, mifepristone; UA, ulipristal acetate.

## Data Availability

All data involved in this study will be made available by the corresponding author upon request.

## Supplementary Materials

The following supporting information can be downloaded at: https://www.mdpi.com/article/10.3390/jcm13113302/s1, Table S1: Risk of bias; Figure S1: Forest plot—changes in dysmenorrhea: GnRHa vs. others; Figure S2: Forest plot—changes in dysmenorrhea: placebo vs. others; Figure S3: Forest plot—changes in uterine volume: LNG-IUS vs. others; Figure S4: Forest plot—changes in uterine volume: dienogest vs. others; Figure S5: Forest plot—changes in uterine volume: COC vs. others; Figure S6: Forest plot—changes in bleeding patterns: LNG-IUS vs. others; Figure S7: Forest plot—changes in bleeding patterns: DNG vs. others; File S1: PRISMA check list.
