# Supplementary material for: The Role of Different Medical Therapies in the Management of Adenomyosis: A Systematic Review and Meta-Analysis
Source: J Clin Med. 2024 Jun 4;13(11):3302. doi: 10.3390/jcm13113302 (PMC11172524; doi:10.3390/jcm13113302)
Supplement: Supplementary file 1 [file jcm-13-03302-s001.zip › jcm-2972490-supplementary.pdf]

Supplementary materials

Table S1 - Risk of bias.

Figure S1: Forest plot - changes in dysmenorrhea GnRHa vs Others;

Figure S2: Forest plot - changes in dysmenorrhea placebo vs Others;

Figure S3: Forest plot - changes in uterine volume LNG-IUS vs Others;

Figure S4: Forest plot - changes in uterine volume Dienogest vs Others;

Figure S5: Forest plot - changes in uterine volume COC vs Others;

Figure S6: Forest plot - changes in bleeding patterns LNG-IUS vs Others;

Figure S7: Forest plot - changes in bleeding patterns DNG vs Others.

File S1: PRISMA check list

Table S1: Risk of bias

Author and year	Risk of bias							
	Bias due to confounding	Bias in selection of participants into the study	Bias in classification of interventions	Bias due to deviations from intended interventions	Bias due to missing data	Bias in measurements of outcomes	Bias in selection of the reported result	Overall risk of bias
Badaway et al. 2012	Low	Low	Low	Low	Low	Moderate	Low	Moderate
Fawzi et al. 2015	Low	Low	Low	Low	Low	Moderate	Low	Moderate
Shaaban et al. 2015	Low	Low	Low	Low	Moderate	Moderate	Low	Moderate
Osuga et al. 2017	Low	Low	Low	Low	Low	Moderate	Low	Moderate
Li et al. 2018	Low	Low	Low	Low	Low	Low	Moderate	Moderate
Matsushima et al. 2018	Low	Moderate	Low	Low	Low	Moderate	Low	Moderate
Hassanin et al. 2020	Low	Low	Low	Low	Low	Moderate	Low	Moderate
Capmas et al. 2021	Low	Moderate	Low	Low	Low	Moderate	Low	Moderate
Ota et al. 2021	Low	Low	Low	Low	Moderate	Moderate	Low	Moderate
Che et al. 2023	Low	Low	Low	Low	Low	Low	Low	Moderate
Guo et al. 2023	Low	Low	Low	Low	Low	Low	Moderate	Moderate
Choundhury et al. 2024	Low	Low	Low	Low	Low	Moderate	Low	Moderate

	GnRHa			others				Mean Difference		Mean Difference
Study or Subgroup	Mean	SD	Total	Mean	SD	Total	Weight	IV, Random, 95% CI	Year	IV, Random, 95% CI
Fawzy 2015	-3.47	1.86	19	-4	2.49	22	48.2%	0.53 [-0.80, 1.86]	2015	
Li 2018	-7.25	0.92	40	-4.29	0.56	40	51.8%	-2.96 [-3.29, -2.63]	2018	
**Total (95% CI)**			**59**			**62**	**100.0%**	**-1.28 [-4.70, 2.14]**		

Heterogeneity: Tau² = 5.84; Chi² = 24.71, df = 1 (P < 0.00001); I² = 96%
Test for overall effect: Z = 0.73 (P = 0.46)

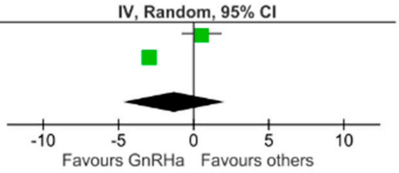

Figure S1: Forest plot - changes in dysmenorrhea GnRHa vs Others;

Study or Subgroup	Placebo Mean	Placebo SD	Placebo Total	others Mean	others SD	others Total	Weight	Mean Difference IV, Random, 95% CI	Year
Osuga 2017	-1.4	1.8	33	-3.8	1.9	34	34.1%	2.40 [1.51, 3.29]	2017
Capmas 2021	-1.5	2.5	10	-1.5	2.5	30	31.3%	0.00 [-1.79, 1.79]	2021
Che 2023	-0.95	1.75	65	-6.63	1.92	61	34.6%	5.68 [5.04, 6.32]	2023
**Total (95% CI)**			**108**			**125**	**100.0%**	**2.78 [-0.25, 5.82]**	

Heterogeneity: Tau² = 6.82; Chi² = 57.39, df = 2 (P < 0.00001); I² = 97%
Test for overall effect: Z = 1.80 (P = 0.07)

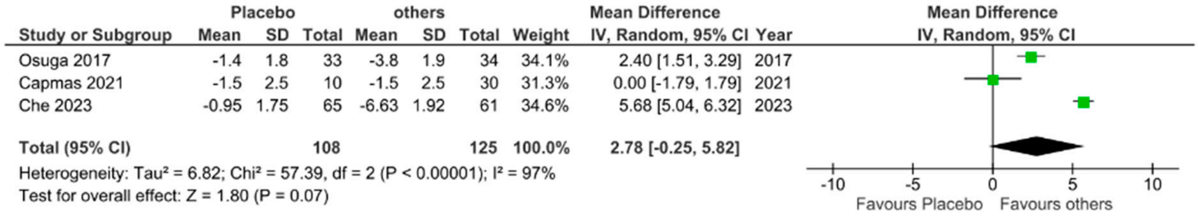

Figure S2: Forest plot - changes in dysmenorrhea placebo vs Others

	LNG-IUS			Others				Mean Difference	
Study or Subgroup	Mean	SD	Total	Mean	SD	Total	Weight	IV, Random, 95% CI	Year
Shaaban 2015	-2.6	1.16	31	-2.1	1.34	31	34.1%	-0.50 [-1.12, 0.12]	2015
Li 2018	20.5	13.12	40	-72	32.7	40	33.9%	92.50 [81.58, 103.42]	2018
Guo 2023	22	80.1	48	5.2	99.7	79	32.0%	16.80 [-14.77, 48.37]	2023
**Total (95% CI)**			**119**			**150**	**100.0%**	**36.53 [-34.53, 107.60]**	

Heterogeneity: Tau² = 3850.35; Chi² = 278.89, df = 2 (P < 0.00001); I² = 99%
Test for overall effect: Z = 1.01 (P = 0.31)

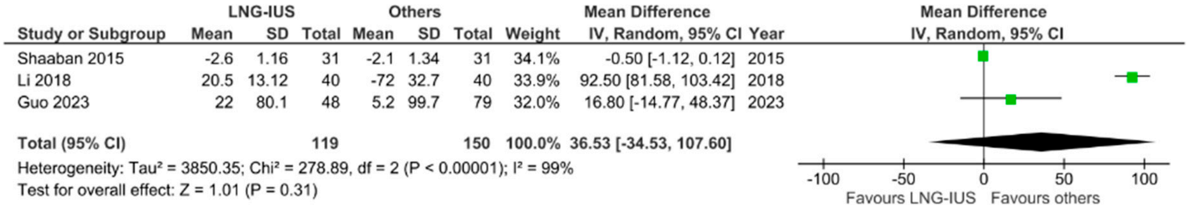

Figure S3: Forest plot - changes in uterine volume LNG-IUS vs Others

	Dienogest			others				Mean Difference	
Study or Subgroup	Mean	SD	Total	Mean	SD	Total	Weight	IV, Random, 95% CI	Year
Fawzy 2015	-33	202.56	22	-3.47	199	19	2.6%	-29.53 [-152.70, 93.64]	2015
Osuga 2017	-20	28.8	34	-9.6	23	33	37.6%	-10.40 [-22.86, 2.06]	2017
Matsushima 2018	29.5	180.5	11	-129.5	270.44	26	1.8%	159.00 [10.06, 307.94]	2018
Hassanin 2020	-42.44	26.86	55	-10.13	43.65	55	36.7%	-32.31 [-45.85, -18.77]	2020
Guo 2023	5.2	99.7	79	22	80.1	48	21.3%	-16.80 [-48.37, 14.77]	2023
**Total (95% CI)**			**201**			**181**	**100.0%**	**-17.26 [-37.62, 3.10]**	

Heterogeneity: Tau² = 246.41; Chi² = 11.05, df = 4 (P = 0.03); I² = 64%
Test for overall effect: Z = 1.66 (P = 0.10)

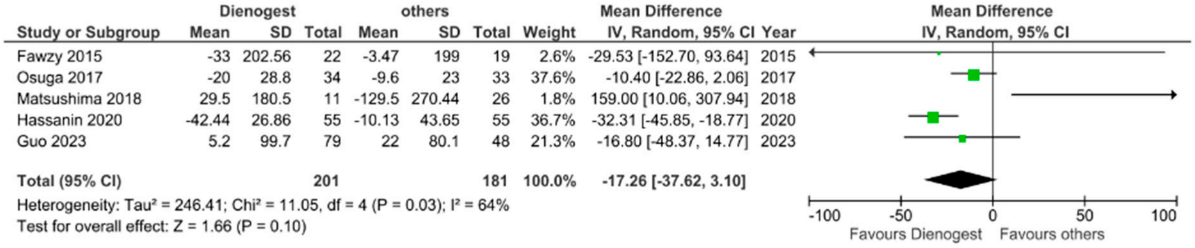

Figure S4: Forest plot - changes in uterine volume Dienogest vs Others

Study or Subgroup	COC Mean	COC SD	COC Total	others Mean	others SD	others Total	Weight	Mean Difference IV, Random, 95% CI	Year
Shaaban 2015	-2.1	1.34	31	-2.6	1.16	31	49.5%	0.50 [-0.12, 1.12]	2015
Matsushima 2018	3.8	173.6	15	-129.5	270.44	26	5.0%	133.30 [-2.80, 269.40]	2018
Hassanin 2020	-10.13	43.65	55	-42.44	26.86	55	45.5%	32.31 [18.77, 45.85]	2020
**Total (95% CI)**			**101**			**112**	**100.0%**	**21.60 [-10.46, 53.65]**	

Heterogeneity: Tau² = 540.16; Chi² = 24.80, df = 2 (P < 0.00001); I² = 92%

Test for overall effect: Z = 1.32 (P = 0.19)

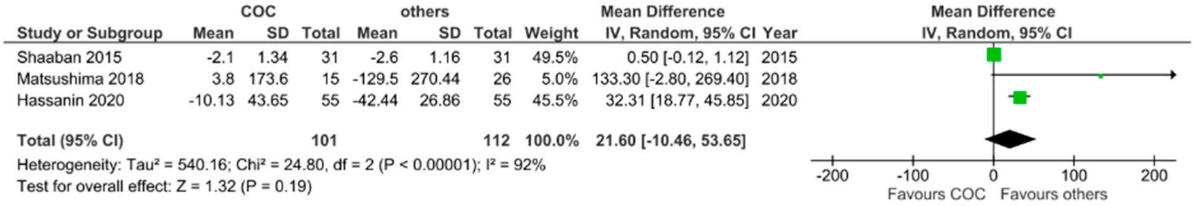

Figure S5: Forest plot - changes in uterine volume COC vs Others

	LNG-IUS			Others				Mean Difference	
Study or Subgroup	Mean	SD	Total	Mean	SD	Total	Weight	IV, Random, 95% CI	Year
Shaaban 2015	-7.18	2.8	31	-2.65	0.87	31	50.0%	-4.53 [-5.56, -3.50]	2015
Ota 2021	5.6	3.41	76	-0.2	3.94	81	50.0%	5.80 [4.65, 6.95]	2021
**Total (95% CI)**			**107**			**112**	**100.0%**	**0.63 [-9.49, 10.75]**	

Heterogeneity: Tau² = 53.04; Chi² = 171.57, df = 1 (P < 0.00001); I² = 99%

Test for overall effect: Z = 0.12 (P = 0.90)

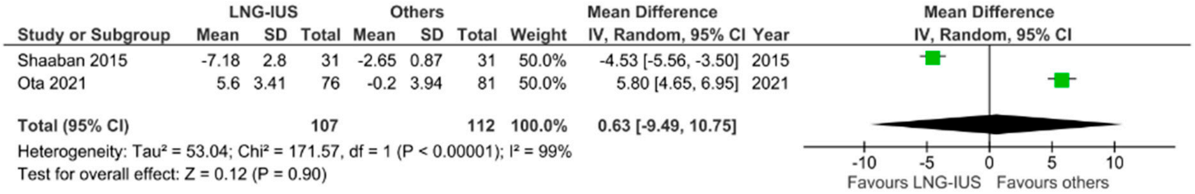

Figure S6: Forest plot - changes in bleeding patterns LNG-IUS vs Others

	Dienogest			others				Mean Difference		Mean Difference
Study or Subgroup	Mean	SD	Total	Mean	SD	Total	Weight	IV, Random, 95% CI	Year	IV, Random, 95% CI
Hassanin 2020	-4.01	3.29	55	-2.25	2.38	55	50.1%	-1.76 [-2.83, -0.69]	2020	
Ota 2021	-0.2	3.94	81	5.6	3.41	76	49.9%	-5.80 [-6.95, -4.65]	2021	
**Total (95% CI)**			**136**			**131**	**100.0%**	**-3.77 [-7.73, 0.18]**		

Heterogeneity: Tau² = 7.84; Chi² = 25.33, df = 1 (P < 0.00001); I² = 96%
Test for overall effect: Z = 1.87 (P = 0.06)

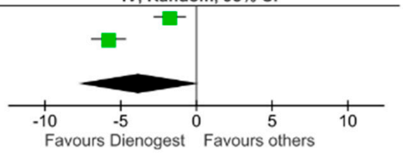

Figure S7: Forest plot - changes in bleeding patterns DNG vs Others

PRISMA check list

PRISMA 2020 Checklist

Section and Topic	Item #	Checklist item	Location where item is reported
**TITLE**			
Title	1	Identify the report as a systematic review.	Page 1, line 3
**ABSTRACT**			
Abstract	2	See the PRISMA 2020 for Abstracts checklist.	Page 1, lines 12-30
**INTRODUCTION**			
Rationale	3	Describe the rationale for the review in the context of existing knowledge.	Page 2, lines 60-75
Objectives	4	Provide an explicit statement of the objective(s) or question(s) the review addresses.	Page 2, lines 75-78
**METHODS**			
Eligibility criteria	5	Specify the inclusion and exclusion criteria for the review and how studies were grouped for the syntheses.	Pages 3, lines 96-99
Information sources	6	Specify all databases, registers, websites, organisations, reference lists and other sources searched or consulted to identify studies. Specify the date when each source was last searched or consulted.	Page 3, lines102-105
Search strategy	7	Present the full search strategies for all databases, registers and websites, including any filters and limits used.	Page 3, lines 109-110
Selection process	8	Specify the methods used to decide whether a study met the inclusion criteria of the review, including how many reviewers screened each record and each report retrieved, whether they worked independently, and if applicable, details of automation tools used in the process.	Page 3, lines 114-120
Data collection process	9	Specify the methods used to collect data from reports, including how many reviewers collected data from each report, whether they worked independently, any processes for obtaining or confirming data from study investigators, and if applicable, details of automation tools used in the process.	Pages 3-4, lines 137-144
Data items	10a	List and define all outcomes for which data were sought. Specify whether all results that were compatible with each outcome domain in each study were sought (e.g. for all measures, time points, analyses), and if not, the methods used to decide which results to collect.	Page 3, lines 123-135
	10b	List and define all other variables for which data were sought (e.g. participant and intervention characteristics, funding sources). Describe any assumptions made about any missing or unclear information.	Page 3, lines 123-135
Study risk of bias assessment	11	Specify the methods used to assess risk of bias in the included studies, including details of the tool(s) used, how many reviewers assessed each study and whether they worked independently, and if applicable, details of automation tools used in the process.	Page 4, lines 147-154
Effect measures	12	Specify for each outcome the effect measure(s) (e.g. risk ratio, mean difference) used in the synthesis or presentation of results.	Page 4, lines 155-164
Synthesis methods	13a	Describe the processes used to decide which studies were eligible for each synthesis (e.g. tabulating the study intervention characteristics and comparing against the planned groups for each synthesis (item #5)).	Page 7 lines 196-200
	13b	Describe any methods required to prepare the data for presentation or synthesis, such as handling of missing summary statistics, or data conversions.	Page 8, lines 231-234
	13c	Describe any methods used to tabulate or visually display results of individual studies and syntheses.	Not applicable
	13d	Describe any methods used to synthesize results and provide a rationale for the choice(s). If meta-analysis was performed, describe the model(s), method(s) to identify the presence and extent of statistical heterogeneity, and software package(s) used.	Page 4, lines 155-164
	13e	Describe any methods used to explore possible causes of heterogeneity among study results (e.g. subgroup analysis, meta-regression).	Not applicable
	13f	Describe any sensitivity analyses conducted to assess robustness of the synthesized results.	Not applicable
Reporting bias assessment	14	Describe any methods used to assess risk of bias due to missing results in a synthesis (arising from reporting biases).	Not applicable
Certainty assessment	15	Describe any methods used to assess certainty (or confidence) in the body of evidence for an outcome.	Not applicable

PRISMA 2020 Checklist

Section and Topic	Item #	Checklist item	Location where item is reported
**TITLE**			
Title	1	Identify the report as a systematic review.	Page 1, line 3
**ABSTRACT**			
Abstract	2	See the PRISMA 2020 for Abstracts checklist.	Page 1, lines 12-30
**INTRODUCTION**			
Rationale	3	Describe the rationale for the review in the context of existing knowledge.	Page 2, lines 60-75
Objectives	4	Provide an explicit statement of the objective(s) or question(s) the review addresses.	Page 2, lines 75-78
**METHODS**			
Eligibility criteria	5	Specify the inclusion and exclusion criteria for the review and how studies were grouped for the syntheses.	Pages 3, lines 96-99
Information sources	6	Specify all databases, registers, websites, organisations, reference lists and other sources searched or consulted to identify studies. Specify the date when each source was last searched or consulted.	Page 3, lines102-105
Search strategy	7	Present the full search strategies for all databases, registers and websites, including any filters and limits used.	Page 3, lines 109-110
Selection process	8	Specify the methods used to decide whether a study met the inclusion criteria of the review, including how many reviewers screened each record and each report retrieved, whether they worked independently, and if applicable, details of automation tools used in the process.	Page 3, lines 114-120
Data collection process	9	Specify the methods used to collect data from reports, including how many reviewers collected data from each report, whether they worked independently, any processes for obtaining or confirming data from study investigators, and if applicable, details of automation tools used in the process.	Pages 3-4, lines 137-144
Data items	10a	List and define all outcomes for which data were sought. Specify whether all results that were compatible with each outcome domain in each study were sought (e.g. for all measures, time points, analyses), and if not, the methods used to decide which results to collect.	Page 3, lines 123-135
	10b	List and define all other variables for which data were sought (e.g. participant and intervention characteristics, funding sources). Describe any assumptions made about any missing or unclear information.	Page 3, lines 123-135
Study risk of bias assessment	11	Specify the methods used to assess risk of bias in the included studies, including details of the tool(s) used, how many reviewers assessed each study and whether they worked independently, and if applicable, details of automation tools used in the process.	Page 4, lines 147-154
Effect measures	12	Specify for each outcome the effect measure(s) (e.g. risk ratio, mean difference) used in the synthesis or presentation of results.	Page 4, lines 155-164
Synthesis methods	13a	Describe the processes used to decide which studies were eligible for each synthesis (e.g. tabulating the study intervention characteristics and comparing against the planned groups for each synthesis (item #5)).	Page 7 lines 196-200
	13b	Describe any methods required to prepare the data for presentation or synthesis, such as handling of missing summary statistics, or data conversions.	Page 8, lines 231-234
	13c	Describe any methods used to tabulate or visually display results of individual studies and syntheses.	Not applicable
	13d	Describe any methods used to synthesize results and provide a rationale for the choice(s). If meta-analysis was performed, describe the model(s), method(s) to identify the presence and extent of statistical heterogeneity, and software package(s) used.	Page 4, lines 155-164
	13e	Describe any methods used to explore possible causes of heterogeneity among study results (e.g. subgroup analysis, meta-regression).	Not applicable
	13f	Describe any sensitivity analyses conducted to assess robustness of the synthesized results.	Not applicable
Reporting bias assessment	14	Describe any methods used to assess risk of bias due to missing results in a synthesis (arising from reporting biases).	Not applicable
Certainty assessment	15	Describe any methods used to assess certainty (or confidence) in the body of evidence for an outcome.	Not applicable
